# Supplementary material for: Case-Fatality and Temporal Trends in Patients with Psoriasis and End-Stage Renal Disease
Source: J Clin Med. 2022 Jul 26;11(15):4328. doi: 10.3390/jcm11154328 (PMC9368806; doi:10.3390/jcm11154328)
Supplement: Supplementary file 1 [file jcm-11-04328-s001.zip › jcm-1762741-supplementary.pdf]

Table S1. Baseline characteristics, treatment and outcome of patients with end-stage renal disease stratified by concomitant psoriasis (n = 1063) with and without psoriatic arthritis (cumulative data of the years 2005–2020).

Parameters	ESRD and Psoriasis with Psoriatic Arthritis (n = 98; 9.2%)	ESRD and Psoriasis without Psoriatic Arthritis (n = 965; 90.8%)	Absolute Risk Difference (95%CI)
Age (years)	66 (56–75)	66 (56–75)	-
Sex (female)	48 (52.2%)	313 (32.4%)	+16·0% (+6·4%; +26·7%)
Obesity	19 (20.7%)	168 (17.4%)	+1·9% (−5·1%; +11·2%)
In-hospital stay	9 (3–20)	8 (3–16)	–
**Comorbidities**			
Coronary artery disease	38 (41.3%)	295 (30.6%)	+8·2% (−1·3%; +18·5%)
Malignancy	5 (5.4%)	47 (4.9%)	+0·2% (−3·1%; +6·6%)
Left heart failure	26 (28.3%)	263 (27.3%)	+0·7% (−8·9%; +9·1%)
Right heart failure	11 (11.9%)	103 (10.7%)	+0·5% (−4·7%; +8·5%)
COPD	14 (15.2%)	161 (16.7%)	−2·4% (−8·5%; +6·2%)
Diabetes mellitus	42 (45.6%)	417 (43.2%)	−0·4% (−10·2%; +10·0%)
Arterial hypertension	48 (52.1%)	488 (50.6%)	−1·6% (−11·7%; +8·6%)
Hypertensive Nephropathy	11 (11.9%)	92 (9.5%)	+1·7% (−3·6%; +9·6%)
Atrial fibrillation/-flutter	19 (20.7%)	222 (23.0%)	−3·6% (−10·8%; +5·7%)
Deep vein thrombosis or thrombophlebitis	n. c.	7 (0.7%)	–
**Dialysis modalities**			
Dialysis general	44 (47.8%)	430 (44.6%)	−0·3% (−9·6%; +10·7%)
Haemofiltration	n. c.	24 (2.5%)	–
Haemodialysis	42 (45.7%)	424 (43.9%)	−1·8% (−10·8%; +5·7%)
Haemodiafiltration	10 (10.9%)	101 (10.5%)	−0·1% (−5·3%; +7·5%)
**Adverse events during hospitalization**			
Gastro-intestinal bleeding	n. c.	5 (0.0%)	–
Intracranial bleeding	0 (0.0%)	0 (0.0%)	0·0% (−0·0%; +3·8%)
Myocardial infarction	n. c.	12 (1.2%)	–
Ischemic Stroke	0 (0.0%)	11 (1.1%)	−0·1% (−20·3%; +2·7%)
Pulmonary embolism	n. c.	5 (0.5%)	–
Cardio-pulmonary reanimation	n. c.	13 (1.3%)	–
Transfusion of erythrocytes	25 (27.2%)	189 (19.6%)	−5·9% (−2·1%; +15·7%)
MACCE	7 (7.6%)	68 (7.0%)	−0·1% (−3·9%; +7·1%)
In-hospital mortality	6 (6.5%)	53 (5.5%)	−0·6% (−3·1%; +7·4%)

n.c. = not calculable
